# Characterization and Functional Divergence of a Novel *DUF668* Gene Family in Rice Based on Comprehensive Expression Patterns

**DOI:** 10.3390/genes10120980

**Published:** 2019-11-28

**Authors:** Hua Zhong, Hongyu Zhang, Rong Guo, Qiang Wang, Xiaoping Huang, Jianglin Liao, Yangsheng Li, Yingjin Huang, Zhaohai Wang

**Affiliations:** 1 State Key Laboratory for Hybrid Rice, College of Life Sciences, Wuhan University, Wuhan 430072, China; 2 Key Laboratory of Crop Physiology, Ecology and Genetic Breeding (Jiangxi Agricultural University), Ministry of Education of the P.R. China, Nanchang 330045, China; 3 Southern Regional Collaborative Innovation Center for Grain and Oil Crops in China, Changsha 410128, China

**Keywords:** *DUF668* gene family, rice, phylogenetic analysis, expression patterns, stress

## Abstract

The domain of unknown function (DUF) superfamily encodes proteins of unknown functions in plants. Among them, *DUF668* family members in plants possess a 29 amino-acid conserved domain, and this family has not been described previously. Here, we report this plant-specific novel *DUF668* gene family containing 12 *OsDUF668* genes in rice (*Oryza sativa*) and 91 *DUF668*s for the other seven plant species. In our study, *DUF668* genes were present in both dicot and monocot plants, indicating that *DUF668* is a conserved gene family that originated by predating the dicot–monocot divergence. Based on the gene structure and motif composition, the *DUF668* family consists of two distinct clades, I and II in the phylogenetic tree. Remarkably, *OsDUF668* genes clustered on the chromosomes merely show close phylogenetic relationships, suggesting that gene duplications or collinearity seldom happened. *Cis*-elements prediction display that over 80% of *DUF668s* contain phytohormone and light responsiveness factors. Further comprehensive experimental analyses of the *OsDUF668* family are implemented in 22 different tissues, five hormone treatments, seven environmental factor stresses, and two pathogen-defense related stresses. The *OsDUF668* genes express ubiquitously in analyzed rice tissues, and seven genes show tissue-specific high expression profiles. All *OsDUF668s* respond to drought, and some of Avr9/Cf-9 rapidly elicited genes resist to salt, wound, and rice blast with rapidly altered expression patterns. These findings imply that *OsDUF668* is essential for drought-enduring and plant defense. Together, our results bring the important role of the *DUF668* gene family in rice development and fitness to the fore.

## 1. Introduction

The structures, functions, and evolution patterns of gene families have hitherto long fascinated plant biologists. Our understanding of the interaction and adaptation to plants with the environment is based on the vast amount of information available for these gene families [1]. Among them, the domain of unknown function (DUF) families refers to a certain kind of protein families with domains of unknown function. Generally, DUF families consist of numerous members, and the entire numbering scheme has extended to *DUF2607* from 1997 to 2010 [2]. Multiple attempts have been made to investigate several DUF genes experimentally. For instance, the *DUF1* domain (*GGDEF*) was isolated and detected to stimulate c-di-GMP (cyclic di-guanosine monophosphate) levels as cyclase, and *DUF2* (*EAL*) degrades c-di-GMP as phosphodiesterase conversely in prokaryotes [3]. *DUF27*, known as the macro domain, possesses the ability to bind the ADP-ribose specifically [4]. Moreover, the *DUF283* domain of Dicer proteins was required for siRNA processing in gene silence [5,6]. The *DUF538* superfamily was predicted to perform hydrolytic activity towards chlorophyll molecules [7,8]. In addition, the amaranth abiotic stress-induced gene (*AhDGR2*) encodes a *DUF642* protein involved in salt and abscisic acid (ABA) hyper-sensibility in *Arabidopsis* [9]. The *DUF1110* was crystallized and identified as the *OsPUB44*-interacting protein 1 (*PBI1*) in rice [10]. The structure of *DUF1470* analysis revealed an ABATE domain (PF07336) and suggested its function presumably as the stress-induced transcriptional regulator [11]. Some members of the monocot-specific *DUF1618* superfamily were reported to participate in chloroplast biogenesis and photosynthesis pathways [12].

Although some DUF gene families have been characterized, a large set of the DUF members remains unknown, especially in rice, a model monocotyledon benefited from its small genomic size and economic importance. *DUF668* domain-containing genes were identified to contain a conserved domain of 29 amino acids (aa). However, there is limited research about this gene family. In plant-pathogen interactions, a dominant or semidominant resistance (R) gene in plant confers resistance to the corresponding avirulence (Avr) peptides from pathogens [13]. Cf-9 is a protein conferring resistance to specific races of tomato *C.fulvum* through recognition of Avr peptides, and the Avr9/Cf-9 recognition to pathogen invasion has served as an excellent model system to dissect the signaling pathways of plant defense responses [14]. Many signaling components acting downstream of Cf-9, Avr9/Cf-9 rapidly elicited genes are found to play an important role in this defense process [15]. Several studies have been performed to elaborate on the disease resistance function of Avr9/Cf-9 rapidly elicited genes in solanaceous plants, such as tomato and tobacco, where the studied Avr9/Cf-9 rapidly elicited genes rapidly respond to Avr9 elicitation and wounding [16,17]. Wounding is usually caused by herbivores and environmental mechanical stresses and opens the way to invasion by microbial pathogens, inducing the plant defense of the dangerous passage [18]. A large number of genes have been found to respond to the wound stress rapidly in *Arabidopsis* [19]. However, it has not been studied whether Avr9/Cf-9 rapidly elicited genes in rice respond to the pathogen-defense related stresses like wounds, and even to any other stresses. It will be interesting to explore the possible functions of these rice Avr9/Cf-9 rapidly elicited genes.

In this study, we distinguished 12 *DUF668* members in the rice genome, which were subsequently divided into two clades (I and II) based on phylogenetic analysis. The *OsDUF668* genes in clade I were annotated as Avr9/Cf-9 exhibited genes. The expression profiles of *OsDUF668* genes were investigated in 22 different rice tissues, five hormone treatments, seven environmental factor stresses, and two pathogen-defense related stresses. The possible functions of these *OsDUF668* genes were explored and discussed. Our research highlights a novel insight into the evolutionary pattern of the *DUF668* family and addresses a better understanding of their functions in rice. 

## 2. Materials and Methods 

### 2.1. Identification of the DUF668 Genes 

The reference genome sequences of eight plants including *Arabidopsis thaliana* (Araport11), *Brachypodium distachyon* (v3.1), *Oryza sativa* (v7.0), *Panicum virgatum* (v5.1), *Sorghum bicolor* (v3.1.1), *Setaria italica* (v2.2), and *Zea mays* (Ensembl-18) which were retrieved from the Phytozome database (http://phytozome.jgi.doe.gov/pz/portal.html) and *Oryza rufipogpon* (OR_W1943) genome database acquired from Ensembl Plants (http://plants.ensembl.org/). The hidden Markov model (HMM) profile (No. PF05003) was downloaded from Pfam (http://pfam.xfam.org/) [20] and used to identify a proteins database in each species by the hidden Markov model (HMM) in HMMER 3.2.1 software (http://hmmer.org/) [21]. Non-redundant *DUF668* proteins were verified by SMART (Simple Modular Architecture Research Tool) (http://smart.embl-heigelberg.de/), and Pfam individually. We searched China’s National Rice Data Center (http://www.ricedata.cn/gene/) to obtain annotation information of *OsDUF668s*. Finally, physicochemical parameters of the rice *DUF668* proteins including theoretical isoelectric points (pI) and molecular weights (MW), were generated by ProtParam tools [22], and the subcellular localizations were predicted via the Plant-PLoc server (http://www.csbio.sjtu.edu.cn/bioinf/plant-multi/) [23]. The existence of *DUF668* proteins in different organisms was also consulted in the European Bioinformatics Institute database (EMBL-EBI, https://www.ebi.ac.uk/).

### 2.2. Phylogenetic Analysis, Gene Structure, and Conserved Motifs

Multiple amino acid sequences alignments were performed by ClustalW of MEGA 7.0 with default parameters [24]. Unrooted Neighbor-Joining (NJ) phylogenetic tree was constructed using 103 *DUF668* genes in eight plants with 1000 bootstrap replicates. The composition of conserved motifs was searched by the MEME (Multiple Em for Motif Elicitation) online tool (http://meme-suite.org/tools/meme), setting a maximum number to 20 [25]. The TBtools was used to analyze and visualize gene structure (exon/intron) and conserved motifs of *DUF*668 genes [26]. The phylogenetic tree was visualized by Evolview v2 (https://evolgenius.info//evolview-v2/) [27]. 

### 2.3. Chromosomal Locations, Gene Duplication Events, and Cis-acting Elements Analysis

Coordinates on the reference genome sequence of rice *DUF668* genes were obtained from the genome annotation file. Gene duplication events were discriminated by MCScanX software [28]. Locations of *OsDUF668* genes were mapped by TBtools [26]. Also, 2 Kb upstream promoter of each rice *DUF668* coding sequence was examined, and the cis-acting regulatory element analysis was executed through the PlantCARE dataset (http://bioinformatics.psb.ugent.be/webtools/plantcare/html/) [29]. The radar map was generated by R 3.5.1 scripts manually. 

### 2.4. Plant Materials and Treatments 

In our case, tissue samples were collected from the *Oryza sativa* ssp. *indica* cultivar 93–11 under natural field condition. Altogether, 22 tissues were prepared according to the procedure used by Wang et al. 2016 [30], namely root (R, 12-day-old seedlings), leaf (L, 12-day-old seedlings), sink leaf (SL, unexpanded flag leaf before heading stage), sink flag leaf sheath (SiFLS, before heading stage), flag leaf (FL, one week after heading), source flag leaf sheath (SoFLS, one week after heading), node (N, the first node on the top at panicle stage), internode (IN, part between the first node and the second node on the top at panicle stage), panicle (P5, P10, P15, and P20: panicle grown to the length of 5 cm, 10 cm, 15 cm, and 20 cm, respectively), hull of flower (H1, 1–3 days before flowering), stamen (St, 1–3 days before flowering), pistil (Pi, 1–3 days before flowering ), spikelets (Sp1, Sp5, Sp15, Sp20, at 1, 5, 15, and 20 days after flowering), hull of seed (H2, at 12–15 days after flowering), immature seed (IS, embryo and endosperm at 12–15 days after flowering), and calli (Ca, induced 30 days before subculture). Three biological replicates were implemented. Each sample was collected from over three plants and pooled together. Samples were grounded immediately with liquid nitrogen and stored in TRIzol Reagent (Invitrogen) at −80 °C for use.

Different experimental treatments were carried out using 12-day-old 93–11 seedlings growing in vermiculite with sterilized water (26 °C, 16-h light/8-h dark). For hormone treatments, seedlings were sprayed with solutions containing 6-benzyl amino purine (6BA) (25 μM), indole-3-acetic acid (IAA) (50 μM), gibberellic acid (GA) (100 μM), salicylic acid (SA) (100 μM), and abscisic acid (ABA) (100 μM), respectively [30,31]. For two environmental factors, heat of 40 °C (H40) and cold of 4 °C (Cold4), the culture temperature of 12-day-old seedlings was separately changed to 40 °C and 4 °C [30,31]. Samples were collected at 0, 1, 3, 6, 12 h after the application of the above treatments. For submergence (Sub), seedlings were submerged in the water with a depth of 5 cm from leaf top to the water surface, and samples were collected at 0, 12, 24, 48, and 72 h after treatment [30]. Seedling samples were collected at 0, 0.25, 0.5, 1, 3, 6, 12 h for all following treatments. For ultraviolet-B (UV-B), 12-day old seedlings were placed in the 24-h light condition (100 μmol m^−2^ s^−1^) supplementing with continuous irradiation of UV-B (500 mWm−2) [30]. As to NaCl, polyethylene glycol (PEG), and drought (Dr) treatments, 12-day-old seedlings were firstly washed off the vermiculite from the roots, and secondly immersed in NaCl solution (200 mM), PEG6000 solution (20%, w/v), and air, respectively [30,32]. For wound stress, seedling leaves were subjected to manual scissors of about nearly one-third of the leaf from the top [30,33]. For rice blast treatment, seedlings were sprayed with 70 ml spore suspension (about 105 conidia ml^−1^) and inoculated under dark and high-humidity conditions [30,34]. Three biological replicates were performed, with each sample collected from about eight seedlings and pooled together. All samples were grounded immediately with liquid nitrogen, and stored in TRIzol Reagent (Invitrogen) at −80 °C to preserve full-length RNA.

### 2.5. RNA Isolation and RT-PCR Analysis

Total RNAs of samples were isolated using TRIzol Reagent (Invitrogen). The first strand of cDNA was synthesized from 5μg of total RNA using the M-MLV reverse transcriptase (Promega). The quantitative Real-Time PCR (qRT-PCR) was conducted with gene-specific primers (Table S1) in a 96-well plate by Bio-Rad CFX96 real-time PCR system, using 2× SYBR Green Master Mix reagent (Bio-Rad). The thermal cycles were as follows: 95 °C for 5 min; 40 cycles of 95 °C for 10 s, primer-specific annealing temperature for 10 s, and 72 °C for 15 s; and then melt curve from 65 to 95 °C. Reference genes were selected according to each experimental condition, as described in Table S2 [30]. Based on the corresponding reference gene(s), the relative expression levels of *OsDUF668* genes were calculated using the Bio-Rad CFX Manager 2.1 software (tissues), and additionally, log2 (experimental treatments). 

## 3. Results

### 3.1. Genome-Wide Identification and Characterization of DUF668 Genes in Rice

To identify *DUF668* genes in rice, we examined the 29 amino-acid *DUF668* conserved domain through the whole rice genome. As a result, we characterized 12 putative non-redundant entries and named all candidates as *OsDUF668-1* to *OsDUF668-12* according to their chromosomal order, which dispersed unevenly across several chromosomes excluding chromosomes 7–10 (Figure S1). Then, we measured more *OsDUF668*s to acquire more information about their chromosome locations, mRNA length, number of amino acids (aa), MW, and theoretical pI (Table 1). The encoded protein length of those *OsDUF668* genes ranged between 357 and 656 aa; meanwhile, their predicted theoretical pI varied from 6.37 to 10.5. We also predicted the subcellular localization of the rice *DUF668* proteins, of which the majority (7/12) was supposed to locate in the chloroplast, while the others to be targeted to the nucleus, peroxisome, cytoplasm, and mitochondrion (Table 1). Of note, we noticed that *OsDUF668-1*, *-4*, *-5*, *-6*, *-9*, and *-12* were annotated as Avr9/Cf-9 rapidly elicited protein whereas the others were not. In this regard, we tempted to speculate that *DUF668* genes in rice could be clustered into two distinct groups.

### 3.2. Phylogenetic Analysis, Gene Structure, and Motif Composition of the DUF668s in Eight Plants

To extend our understanding of the *DUF668* gene family, we screened out the *DUF668* domain in the European Bioinformatics Institute database (EMBL-EBI, https://www.ebi.ac.uk) and found *DUF668* genes exist in up to 62 plant organisms excluding animal and other non-plant species (Table S3). We further dissected four model genomes (*Homo sapiens*, *Mus musculus*, *Drosophila melanogaster*, and *Danio rerio*) and none DUF668 was found. Thus, this finding manifested DUF668s might belong to a kind of plant-specific gene family. 

**Figure 1:**
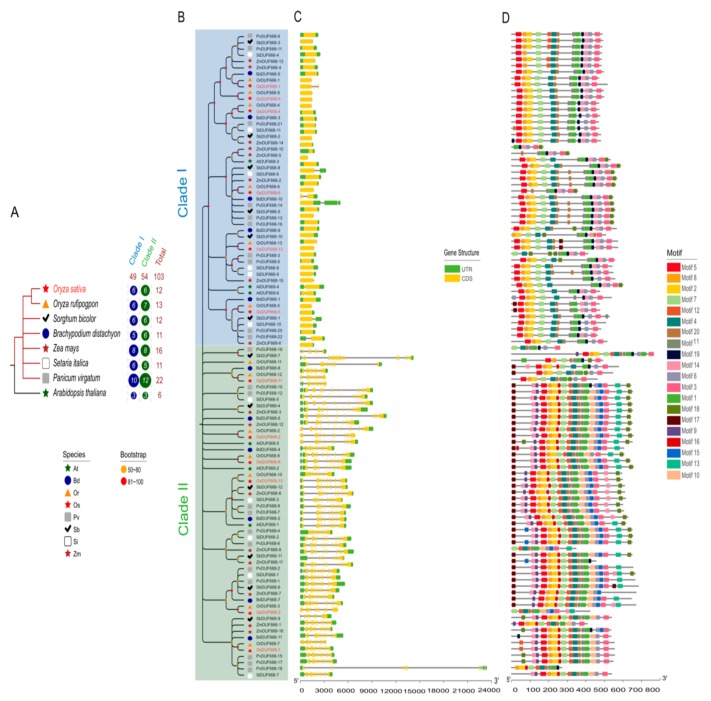
Comparison of the gene structure and motif of 103 *DUF668* genes in eight plant organisms. (**A**) Unrooted phylogenetic tree with over 50% bootstrap value above the branch. The clade I and II are displayed in blue and green colors, separately. The different-shaped arcs indicate different species. The names of species are abbreviated to two letters, namely *Arabidopsis thaliana* (At), *Brachypodium distachyon* (Bd), *Oryza sativa* (Os), *Oryza rufipogpon* (Or), *Panicum virgatum* (Pv), *Sorghum bicolor* (Sb), *Setaria italica* (Si), and *Zea mays* (Zm). (**B**) Motif architectures of all *DUF668* genes. Each motif is illustrated with a specific color, and the distribution of identified motifs corresponds to their positions. (**C**) Exon/introns and untranslated regions (UTRs) of *DUF668s*. Green boxes denote UTR (untranslated region); yellow boxes denote CDS (coding sequence); black lines denote introns. The length of protein can be estimated using the scale at the bottom. (**D**) Evolutionary relationship of the *DUF668* genes from eight plant species. The right circles show the number of *DUF668s* in different plant lineages in relative groups. More detailed inspections are provided in Table S4.

Gramineae is known as the fourth largest flowering plant family, and an essential genetic resource consisted of many commercial crops [35]. Apart from *O. sativa*, we consequently screened *DUF668* members through one model dicot *A. thaliana* genome and six representative graminaceous genomes, including *B. distachyon*, *O. rufipogpon*, *P. virgatum*, *S. bicolor*, *S. italica*, and *Z. mays*. In the above species genomes, 6, 11, 13, 22, 12, 11, and 16 non-redundant *DUF668* family members were detected successively (Figure 1A; Table S4). To investigate the evolutionary relationships of the *DUF668* family in those Gramineae organisms, we conducted a neighbor joining (NJ)-phylogenetic tree combining with exon/intron structural and conserved motifs analysis. The results showed that all 103 *DUF668* proteins could be integrated into the two clades I and II (Figure 1B). Forty-nine and fifty-four *DUF668*s pertained to clade I and II separately, stating a negligible difference in gene copy number. We noticed that half of the rice *OsDUF668*s exhibited in clade I, and all of them were Avr9/Cf-9 mediated genes, which confirmed our preceding assumption. Furthermore, these two distinct groups differentiated not only in gene structure but also in motif arrangement. With a few exceptions, *DUF668*s in clade II had approximately 9–16 exons, in sharp contrast to those in clade I, most of which only possessed one exon (Figure 1C). We identified 20 different conserved motifs (Figure 1D; Table S5). Motif 1, and motif 5 were related to unknown function protein *DUF668* and *DUF3475*, while others have no functional annotation. In general, clade II had more motifs than clade I. Motif 1 was the most common motif, presenting in all *DUF668* genes. Besides that, the vast majority of *DUF668*s included motif 2, 3, 4, 5, 6, 7, 8, and 20. Motif 9, 10, 13, 15, and 16 were group-specific elements in clade II, as motif 19 mainly existed in clade I. To sum up, the exon-intron structures and motif constituent of the *DUF668* genes substantially conformed to their phylogenetic relationships. 

### 3.3. Cis-acting Elements Prediction of DUF668 Genes in Rice

**Figure 2:**
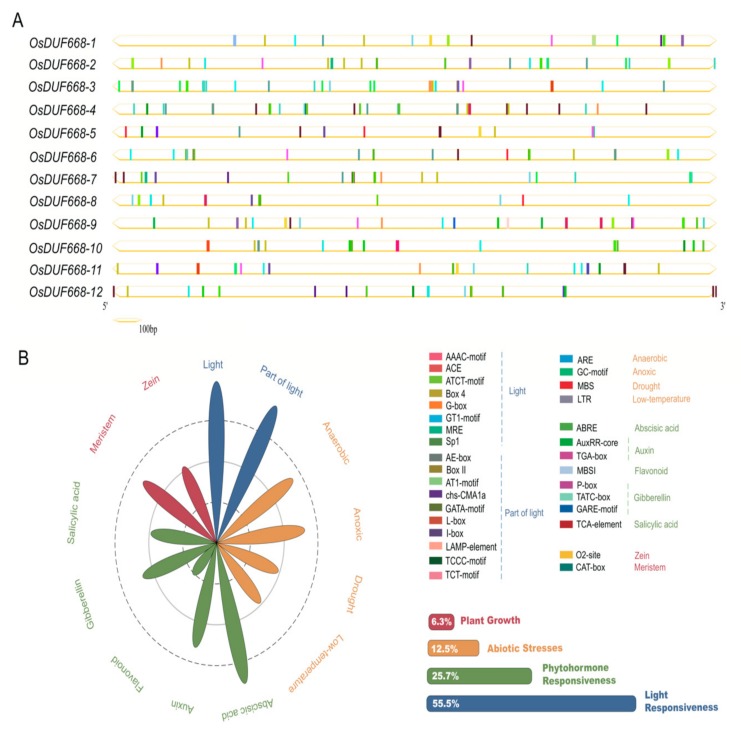
Identification of cis-acting elements in all the *OsDUF668* genes. (**A**) Distribution of cis-acting elements in 2 Kb upstream of each *OsDUF668* gene. The different colored boxes indicate distinct promoter elements. (**B**) Assessment of different subclass and category proportion in a radar chart. The lengths of the petals are proportional to the number of elements in each subclass qualitatively. Red, orange, green, and blue petals represent plant growth regulation, abiotic stress responses, phytohormone and light responsiveness, respectively.

*Cis*-acting regulatory elements might provide clues for determining gene expression patterns in various organs or under environmental stresses [36]. Previous studies reported a significant positive relationship between response genes and their cis-elements in upstream promoter regions [37,38]. Here, we manifested potential cis-regulatory elements in the 2 Kb upstream regions of the *OsDUF668s* examined via the PlantCARE database. Thirty-two cis-regulatory elements were detected totally (Figure 2A), which formed eleven subclasses and four main categories as plant growth, abiotic stress, phytohormone responsiveness, and light responsiveness (Figure 2B). The largest subdivision was light responsiveness, which contained 55.5% predicted *cis*-elements, including AAA-motif (light-responsive element) and AE-box (part of a module for light response) as representatives. A series of regulatory elements participating in plant hormone responsiveness ranked second. Cis-acting factors respond to abscisic acid, auxin, flavonoid, gibberellin, and salicylic acid were involved. Among them, ABRE (related to the abscisic acid response) was covered the largest portion, followed by the TGA-element (auxin-responsive element). In the abiotic stress response category, elements regarding oxygen-deficient induction (GC-motif, ARE) were the most common, followed by those relating to low-temperature responsiveness (LTR) and drought-inducibility (MBS). As for the plant growth regulation category, only two main stress-related cis-acting factors were identified, known as the CAT-box (referred to meristem expression) and O2-site (involved in zein metabolism regulation). Intriguingly, all kinds of *cis*-regulatory elements distributed widely throughout the promoter regions of *OsDUF668* genes, revealing that *OsDUF668s* may have intricate expression profiles and be crucial in the regulation of rice development and stress resistance.

### 3.4. Tissue-Specific Expression Patterns of OsDUF668s

**Figure 3:**
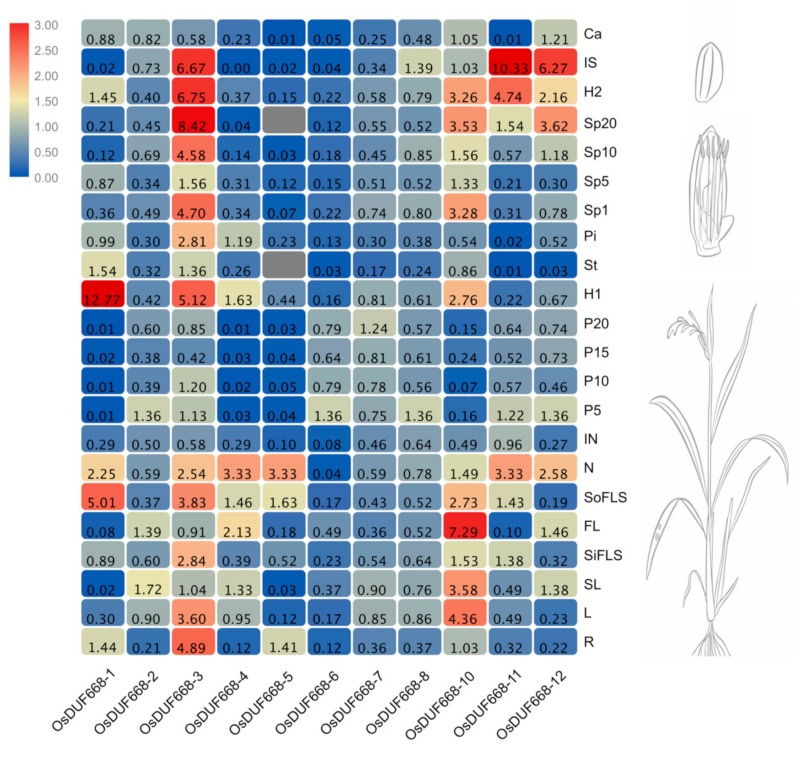
Relative expression levels of *DUF668* genes in rice 22 various tissues. The relative expression levels in the heat map were calculated by the 2^−∆∆CT^ method and represented by the color legend: red indicates high expression level, and blue represents low expression level. The rice morphology diagrams at the right of the heat map were downloaded from Expression Atlas (https://www.ebi.ac.uk/gxa/home).

To further characterize the potential biological function, comprehensive tissue-specific expression analyses of *OsDUF668s* were investigated using qRT-PCR. As displayed in Figure 3, we detected 11 *OsDUF668s* except for the undetectable *OsDUF668-9* in 22 rice tissues involving in various developmental processes. The results showed that these 11 *OsDUF668s* were ubiquitously expressed in 22 rice tissues, except for the undetectable *OsDUF668-5* in anther and spikelets (Sp20, at 20 days after flowering). *OsDUF668-2*, *-6*, *-7*, and *-8* exhibited moderate expression levels and minor expression differences among 22 tissues, except that *OsDUF668-6* expressed negligibly in node (N), internode (IN), anther (An), immature seed (IS), and calli (Ca). Comparatively, the rest *OsDUF668-1*, *-3*, *-4*, *-5*, *-10*, *-11*, and *-12* showed large expression difference among 22 tissues, presenting discernibly, organ-specific high expression patterns. For example, *OsDUF668-1* was negligibly expressed in flag leaf (SL and FL), panicle (P5, P10, P15, and P20), and immature seed (IS), whereas it highly expressed in source flag leaf sheath (SoFLS), node (N) and hull of flower (H1). Additionally, *OsDUF668-3* and *OsDUF668-10* were expressed highly in more than eight different tissues. These tissue expression patterns suggested *OsDUF668s* played an influential role in rice growth and development.

### 3.5. Expression Profiles of OsDUF668s in Response to Plant Hormone

**Figure 4:**
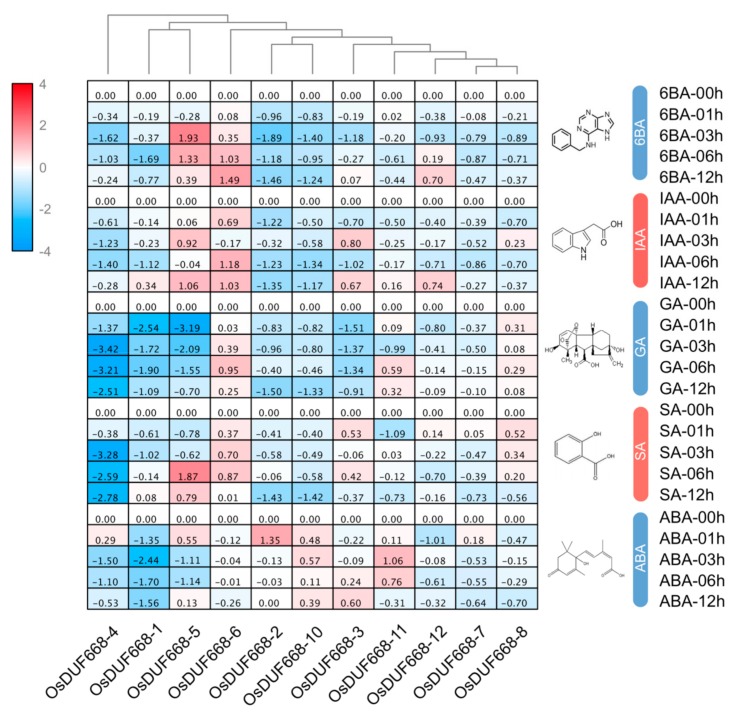
Relative expression of *OsDUF668s* under 6BA, IAA, GA, SA, and ABA treatments. 6BA: 6-benzyl amino purine, IAA: indole-3-acetic acid, GA: gibberellic acid, SA: salicylic acid, and ABA: abscisic acid. Expression characteristics of *OsDUF668s* in response to different phytohormone at four-time points (1, 3, 6, and 12h) were normalized to 0 h treatment. The fold changes values were calculated by the 2^−∆∆CT^ method and log2 and represented in color scale legend at the left of the heat map: red indicated up-regulation and blue showed down-regulated expression.

Plant hormones, including auxin, cytokinin, and ethylene, have a lasting impact on regulating plant architecture and development [39]. In order to explore the possible functions of *OsDUF668s* in response to hormone stress, we used qRT-PCR to analyze their relative expressions under 6BA, IAA, GA, SA, and ABA treatments (Figure 4). In this study, the two-fold change (|log2| > 1) was considered to be a significant difference for the gene expression under each treatment. Of all 11 *OsDUF668s*, seven genes merely up-expressed in any treatment, while the remaining four (*OsDUF668 -2*, *-5*, *-6*, and *-11*) were examined to be elevating expression in at least one treat. For instance, *OsDUF668-5* and *OsDUF668-6* were both up-regulated following 6BA and IAA conditions. Also, *OsDUF668-5* was induced in 3 h treatment of SA. Expression of *OsDUF668-2* and *OsDUF668-11* were elevated when applying with ABA. By contrast, more genes were down-regulated or suppressed for certain hormone treatments, such as *OsDUF668-4* and *OsDUF668-1* under all five hormone conditions, *OsDUF668-2* and *OsDUF668-10* in all five hormone treatments except for ABA, *OsDUF668-3* for 6BA, IAA and GA, and *OsDUF668-5* for GA and ABA. The results indicated that these *OsDUF668* genes might regulate relevant hormone signaling pathways.

### 3.6. Transcriptional Responses of OsDUF668s Facing Environmental Stresses

**Figure 5:**
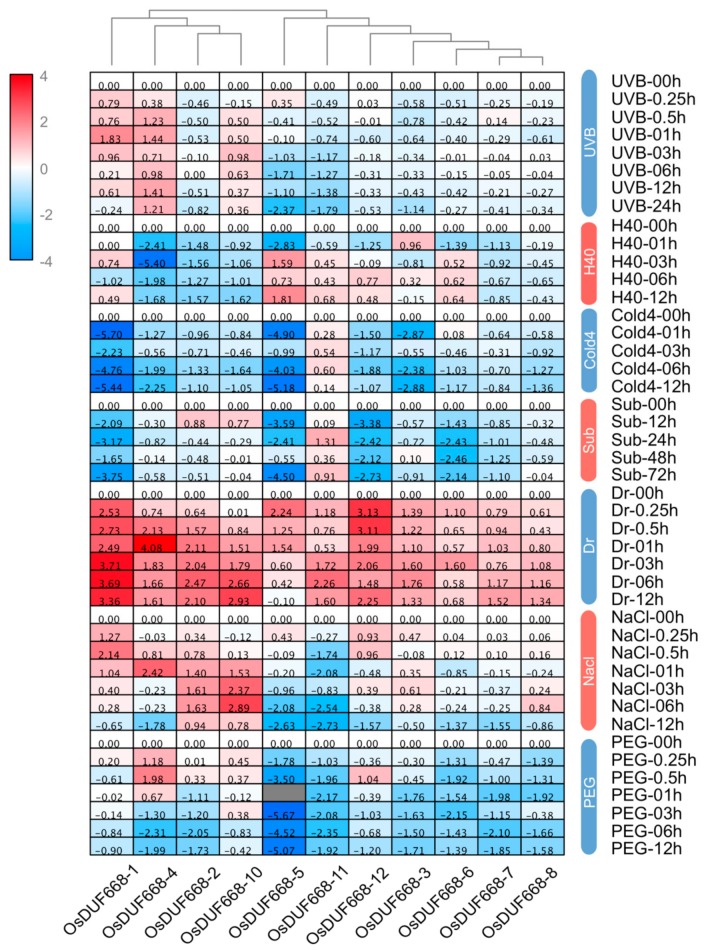
Expression changes of *OsDUF668s* under different environmental stresses. The abbreviation represented as following rules, UVB: ultraviolet-B, H40: the heat of 40 °C, Cold4: cold of 4 °C, Sub: submergence, Dr: drought, and PEG: polyethylene glycol.

Plants suffer from a wide variety of environmental stresses under natural conditions during their life span. As a result, we investigated the expression of *OsDUF668* genes, responding to seven environmental stresses (Figure 5). Results showed the accumulation of *OsDUF668-1,* and *OsDUF668-4* transcripts occurred within 1h after UVB application. Expression levels of *OsDUF668-5* and *OsDUF668-11* were elevated under H40 and Sub conditions, separately. Interestingly, all analyzed *OsDUF668* genes were found to positively respond to the Dr stress, in which, *OsDUF668-1*, *-2*, *-4*, *-5*, *-10*, *-11*, and *-12* showed strong up-regulation of over four times, meanwhile *OsDUF668-1*, *-3*, *-5*, *-6*, *-11* and *-12* showed fast elicitation after the 15-min treatment. Moreover, four *OsDUF668* genes, *OsDUF668-1*, *-2*, *-4*, and *-10* were induced under NaCl stress, and *OsDUF668-1* showed fast elicitation after 15-min stress. Only *OsDUF668-4* was significantly induced for PEG stress. Additionally, many genes were down-regulated or suppressed under environmental stresses, such as *OsDUF668-3*, *-5*, and *-11* for UVB, *OsDUF668-2*, *-4*, *-7*, and *-10* for H40, all *OsDUF668s* but *OsDUF668-*7 or *-11* for Cold4, *OsDUF668-1*, *-5*, *-6*, *-7,* and -*12* for Sub, *OsDUF668-5* and *-11* for NaCl, and *OsDUF668-2*,*- 3*, *-5*, *-6*, *-7*, *-8*, *-11*, and *-12* for PEG stress. In summary, *OsDUF668* genes may act as key roles in various environmental fitness. 

### 3.7. Transcriptional Responses of OsDUF668s Facing Pathogen-Defense Related Stresses

**Figure 6:**
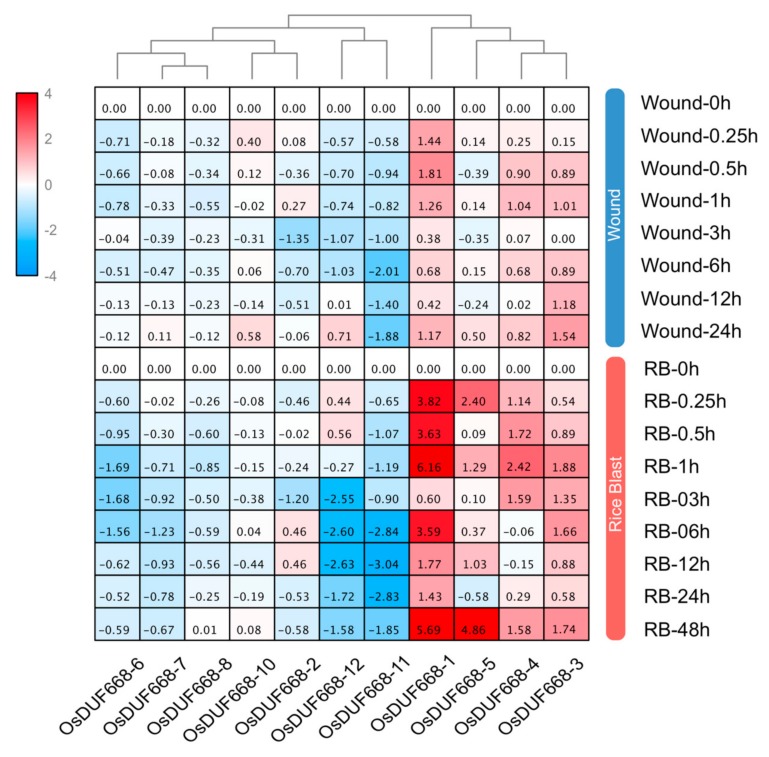
Expression changes of *OsDUF668s* under wound and rice blast stresses. The relative expression levels were showed in the heat map from blue to red. 0h was treated as standard control to normalized data. In the heat map, fold changes reflected relative expression values from blue to red, while gray cells represented undetected values. The abbreviation RB meant rice blast.

Previous studies have reported that Avr9/Cf-9 rapidly elicited genes play an important role in plant defense for pathogen invasion [14,16]. On the fact that half of the *OsDUF668s* were annotated as Avr9/Cf-9 rapidly elicited genes, we implemented qRT-PCR to analyze whether *OsDUF668s* resisted two pathogen-defense related stresses: wound and rice blast (Figure 6). The results showed that *OsDUF668*-*1*, *-3*, *-4*, and *-5* were significantly elevated following wound and rice blast conditions. Transcripts of those genes were strongly induced in particular with rice blast. *OsDUF668-1* showed fast elicitation after 15-min wound stress, meanwhile, *OsDUF668-1*, *-4*, and *-5* exhibited fast elicitation under 15-min rice blast treatment. In addition, expression levels of *OsDUF668-6*, *-7*, *-11*, and *-12* were significantly down-regulated for both pathogen-defense related stresses. The results implied that these *OsDUF668* genes might be involved in rice defense for pathogens. 

## 4. Discussion

In this case, we reported plants to possess a unique set of *DUF668* genes from genome-wide identification profiling. A further study distinguished 103 *DUF668* genes in the databases of dicot *Arabidopsis thaliana* and eight representative crops in Gramineae, explicating that the *DUF668* family originated in an ancestral plant species before the divergence between monocotyledon and dicotyledon (Figure 1). Thereby, *DUF668* has been a conserved gene family for a long evolution. In the monocot grass family (Gramineae), *DUF668* family members in each organism were close in number without regard to *P. virgatum* and *Z. mays*. One alternative explanation may be that the total number of annotated genes from the Phytozome database in rice (52,425) was in the same range as *B. distachyon* (52,973), *S. bicolor* (47,122), *B. distachyon* (52,973), and *S. italica* (43,002). Given that *Z. mays* and *P. virgatum* had relatively large genome size and a great many genes, the number of *DUF668* genes was more than other species consequently. These results indicated similar gene numbers across a broad diversity of economically important grasses. In rice, a multitude of gene family members with high sequence similarity has been observed to cluster on the chromosomes as paralogous pairs [40,41]. However, we identified 12 *OsDUF668s* and found there were no collinearity relationships among them. It meant that the *DUF668* family has still not experienced gene expansion events driven by tandem and segmental duplication. Furthermore, phylogenetic analysis categorized all *DUF668* proteins into two distinct lineages differing from the exon-intron association and motif arrangement (Figure 1). All clade I members in rice were the Avr9/Cf-9 mediated protein without sufficient research. 

*Cis*-acting elements of light, phytohormone, and abiotic stresses responsiveness presented ubiquitously in the promoter region of *OsDUF668*, indicating these optical and chemical impact factors may interact to act on the *DUF668* regulatory mechanism (Figure 2). Tissue expression profiles showed that the majority of *OsDUF668s* were ubiquitously expressed in various developmental stages (Figure 3), implying their roles in the whole life cycle of rice. *OsDUF668-1, -3, -4*, *-5*, *-10*, *-11*, and *-12* also exhibited high expression levels in some specific tissues (Figure 3), reflecting the functional tissue specificity of these *OsDUF668* genes. The positive response of most *OsDUF668* genes to hormones was marginal, regardless of that *OsDUF668-5* and *OsDUF668-6* were significantly up-regulated in 6BA, IAA, and SA treatments (Figure 4), implying their relevant roles in these hormone pathways. Besides, *OsDUF668-1* and *OsDUF668-4* positively responded to UVB, elucidating their roles in rice adaption to the solar UV light (Figure 5). A surprising finding was all thje *OsDUF668* genes were up-regulated extensively by drought stresses (Figure 5), suggesting that *OsDUF668* may be important for rice resistance to drought. Similarly, *OsDUF668-1*, *-2*, *-4*, *and -10* were found to positively regulated in NaCl stress (Figure 5), reflecting that the physiological function of these genes were also involved in plant salt-tolerance mechanisms. Considering the common positive response of *OsDUF668-1*, *-2*, *-4*, and *-10* under drought and NaCl stresses overexpression of these four genes in rice may be effective methods to engineering plant fitness for drought and salinity conditions. 

Avr9/Cf-9 elicited genes play key roles in plant-pathogen interactions. Expression levels of Avr9/Cf-9 elicited genes are found to be differentially changed during transcriptome profile analysis of resistance induced by burdock fructooligosaccharide in tobacco [42]. The protein kinase ACIK1 is encoded by an Avr9/Cf-9 elicited gene and is essential for full Cf-9-dependent disease resistance in tomato [17]. The F-Box protein ACRE189/ACIF1, encoded by another Avr9/Cf-9 elicited gene, regulates cell death, and defense response activated during pathogen recognition in tobacco and tomato [16]. In our study, clade I members of *OsDUF668* family *OsDUF668-1*, *-4* and *-5* expressed highly, and *OsDUF668-6* and *OsDUF668*-*12* were down-regulated in wound and rice blast treatments (Figure 6), displaying these Avr9/Cf-9 elicited genes may also mediate response to biotic defense in rice. Moreover, *OsDUF668-1*, *-3*, *-4*, and *-5* showed positive response under both wound and rice blast conditions (Figure 6), implying that overexpression of these four genes in rice may help to improve plant defense against pathogens. In addition, a key characteristic of the Avr9/Cf-9 elicited gene is that their expression pattern usually rapidly altered (15 min after elicitation) during the biotic defense response [17]. In rice, corresponding Avr9/Cf-9 elicited genes in *DUF668* gene family also exhibited response to Dr (*OsDUF668-1*, *-5*, *-6*, and *-12*), NaCl (*OsDUF668-1*), wound (*OsDUF668-1*) and rice blast (*OsDUF668-1*, *-*4, and *-5*) in a rapid manner of 15 min (Figure 5 and Figure 6), implying the conservation and similarity of regulation and function of Avr9/Cf-9 elicited genes among plant species. However, why and how these Avr9/Cf-9 elicited genes respond so fast still need further study in the plant.

Importantly, the conserved evolutionary property of *DUF668* genes in the rice genome and the responses of some members to adverse situations reflected their importance for rice growth and resistance to multiple stresses. Tackling the function of the *DUF668*s nexus will require more powerful approaches like transgenic experiments. Taken together, we provide a detailed and multi-level view of the *DUF668* gene family in rice. This study underlies further work to dissect the evolutionary significance of this gene family and simultaneously accelerates functional research of their roles in rice growth and development as well as the stress response. 

## Figures and Tables

**Table 1:** Characteristics of the putative *DUF668* genes in rice.

Gene ID	Gene Name	Clade	Chr	mRNA Length (bp)	Protein (aa)	MW (Da)	Theoretical pI	Predicted Location	Annotation
*LOC_Os01g62670*	*OsDUF668-1*	clade-I	Chr1	2317	516	55952.4	8.31	Chloroplast	avr9/Cf-9 rapidly elicited protein
*LOC_Os01g65330*	*OsDUF668-2*	clade-II	Chr1	7233	656	73065.2	9.86	Nucleus	expressed protein
*LOC_Os02g34650*	*OsDUF668-3*	clade-II	Chr2	3899	422	46119.6	10.36	Chloroplast	expressed protein
*LOC_Os03g16390*	*OsDUF668-4*	clade-I	Chr3	1936	471	52064.3	10.07	Peroxisome	avr9/Cf-9 rapidly elicited protein
*LOC_Os03g64130*	*OsDUF668-5*	clade-I	Chr3	1757	476	52051.3	10.31	Chloroplast	avr9/Cf-9 rapidly elicited protein
*LOC_Os04g08764*	*OsDUF668-6*	clade-I	Chr4	2151	357	38783	7.39	Chloroplast	avr9/Cf-9 rapidly elicited protein
*LOC_Os04g35410*	*OsDUF668-7*	clade-II	Chr4	4154	554	61523.2	6.37	Chloroplast	expressed protein
*LOC_Os05g35530*	*OsDUF668-8*	clade-II	Chr5	6418	641	71108.8	9.98	Cytoplasm	expressed protein
*LOC_Os05g38320*	*OsDUF668-9*	clade-I	Chr5	1490	497	53685	10.21	Chloroplast	avr9/Cf-9 rapidly elicited protein
*LOC_Os06g50220*	*OsDUF668-10*	clade-II	Chr6	5930	598	67106.3	9.14	Nucleus	expressed protein
*LOC_Os11g07840*	*OsDUF668-11*	clade-II	Chr11	3264	462	51730.7	7.38	Mitochondrion	expressed protein
*LOC_Os12g05180*	*OsDUF668-12*	clade-I	Chr12	1970	573	62417.4	10.5	Chloroplast	avr9/Cf-9 rapidly elicited protein

## Supplementary Materials

The following are available online at https://www.mdpi.com/2073-4425/10/12/980/s1, Figure S1: Chromosomal locations of *DUF668* genes in rice. Different colors of *OsDUF668s* represent the members of different subfamilies; Table S1: Primers for qRT-PCR; Table S2: Reference genes for each experimental condition; Table S3: *DUF668* gene family number in 62 plant species; Table S4: Summary of information on *DUF668* genes from seven other plants; Table S5: Annotation of conserved motifs in *DUF668* proteins.
