# Supplementary material for: Characterization and Functional Divergence of a Novel DUF668 Gene Family in Rice Based on Comprehensive Expression Patterns
Source: Genes (Basel). 2019 Nov 28;10(12):980. doi: 10.3390/genes10120980 (PMC6969926; doi:10.3390/genes10120980)
Supplement: Supplementary file 1 [file genes-10-00980-s001.zip › genes-633006-sup/Supplementary/Table S1-S2.docx]

**Table 1.** Primers for qRT-PCR.

| **Primer name** | **Primer sequence 5’-3’ (Forward/Reverse)** | **Amplicon length (bp)** | **Annealing temperature (℃)** | **PCR efficiency (%)** | **R^2^** |
| --- | --- | --- | --- | --- | --- |
| OsDUF668-1 | CAAGGAGGAGTCCCTCTGGAA | 88 | 60 | 99.6 | 0.988 |
|  | ACAGATGCGGCTGAACACG |  |  |  |  |
| OsDUF668-2 | CACCACCAAGGCTCACCAT | 106 | 55 | 105.2 | 0.997 |
|  | CTCAATTCGGGTCAGGTCC |  |  |  |  |
| OsDUF668-3 | CGCTCTATCAAGGACTGCC | 188 | 57 | 91.4 | 0.997 |
|  | CCACTCGCCGACCCATC |  |  |  |  |
| OsDUF668-4 | TCGGCTCCCATCTTTCGGC | 178 | 63 | 92.5 | 0.997 |
|  | CGGCGTCCACCTCGTTGCT |  |  |  |  |
| OsDUF668-5 | ACCAGGGCGCACAAGAT | 224 | 52 | 91.2 | 0.999 |
|  | GAGGACCAGAGGGAGGTTT |  |  |  |  |
| OsDUF668-6 | AGAAGACGGAGGCCGTGATA | 158 | 63 | 99.2 | 0.979 |
|  | CTGACCTTGGACTGATGGACTCTAC |  |  |  |  |
| OsDUF668-7 | AAGAACTTCGTCAGTGCCTCAG | 139 | 60 | 96.8 | 0.997 |
|  | TCTCGCCCATTCGCTAAAC |  |  |  |  |
| OsDUF668-8 | GACTTAACCCGAATCGAAACACT | 140 | 52 | 98.2 | 0.999 |
|  | GACTTGATGGGAGACCTTACACC |  |  |  |  |
| OsDUF668-10 | GAAACTCTTCGCTGGATTCTACC | 114 | 60 | 104.4 | 0.992 |
|  | GCCTGATTTCTTGTTCATTTCG |  |  |  |  |
| OsDUF668-11 | ATGGCTGCATCATTGACATGG | 185 | 60 | 93 | 0.998 |
|  | CCAAGTGGTGCTAATGTTACCG |  |  |  |  |
| OsDUF668-12 | GCTGCATGGTCAGTGGGAG | 140 | 60 | 98.1 | 0.994 |
|  | GCCTTGGAACGGGGAATTG |  |  |  |  |

**Table 2.** Reference genes for each experimental condition.

| **Experimental conditions** | **Reference genes** |
| --- | --- |
| 22 tissue samples | ARF, Profillin-2, TI and UBC |
| 6BA | PtfS and Edf |
| IAA | TI and ARF |
| GA | TI and Profilin-2 |
| SA | TI and ARF |
| ABA | TI and ARF |
| UVB | TI and Profilin-2 |
| H40 | TI and ARF |
| Cold4 | PtfS and ARF |
| Sub | Profilin-2 and Edf |
| Dr | TI and ARF |
| NaCl | Ppcti and TI |
| PEG | Ppcti and UBC |
| Wound | Edf and Profilin-2 |
| RB | UBQ5 and Profilin-2 |
