# Supplementary figures and images for: Characterization and Functional Divergence of a Novel DUF668 Gene Family in Rice Based on Comprehensive Expression Patterns
Source: Genes (Basel). 2019 Nov 28;10(12):980. doi: 10.3390/genes10120980 (PMC6969926; doi:10.3390/genes10120980)

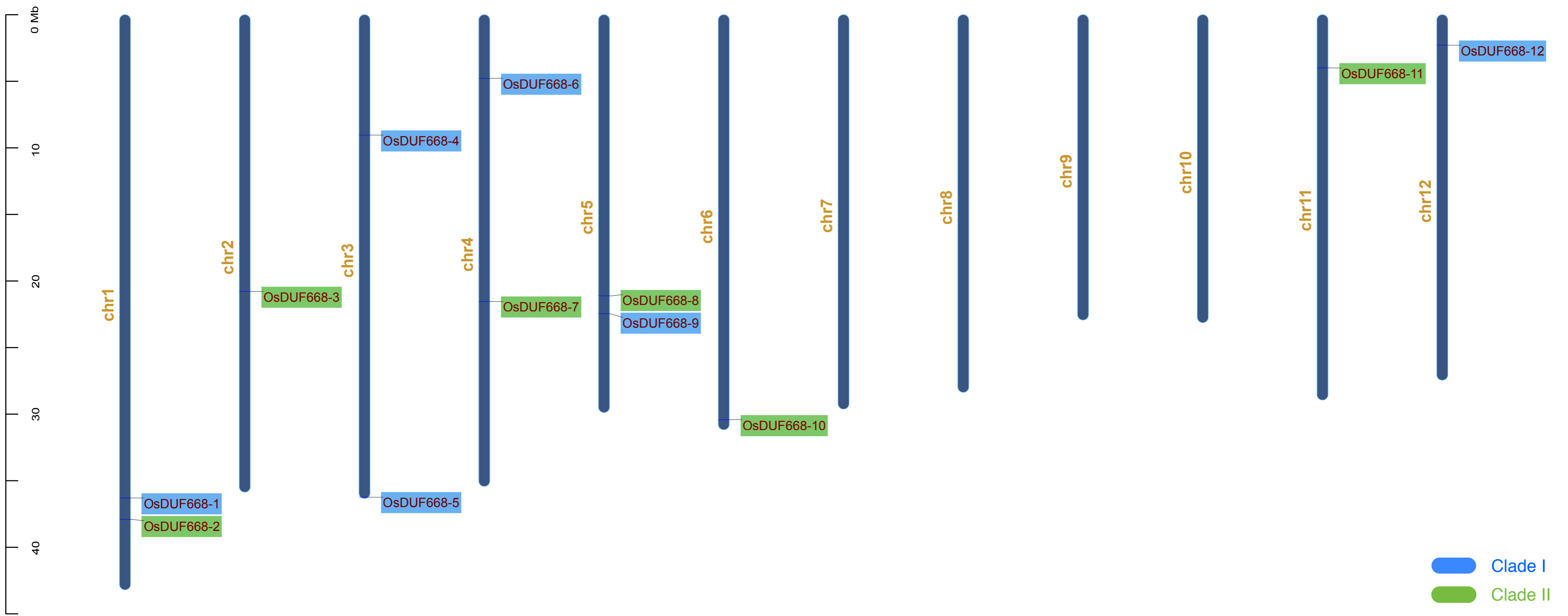

Supplement: Supplementary file 1 [file genes-10-00980-s001.zip › genes-633006-sup/Supplementary/Fig S1.pdf]
